# Electronic cigarette vapor alters the lateral structure but not tensiometric properties of calf lung surfactant

**DOI:** 10.1186/s12931-017-0676-9

**Published:** 2017-11-17

**Authors:** Rebecca J. Przybyla, Jason Wright, Rajan Parthiban, Saeed Nazemidashtarjandi, Savas Kaya, Amir M. Farnoud

**Affiliations:** 1 Biomedical Engineering Program, Department of Chemical and Biomolecular Engineering, 168 Stocker Center, Ohio University, Athens, OH 45701 USA; 2 School of Electrical Engineering and Computer Science, Ohio University, Athens, OH 45701 USA

**Keywords:** Electronic cigarette, Lung surfactant, Surface tension, Infasurf^®^, Surfactant inhibition

## Abstract

**Background:**

Despite their growing popularity, the potential respiratory toxicity of electronic cigarettes (e-cigarettes) remains largely unknown. One potential aspect of e-cigarette toxicity is the effect of e-cigarette vapor on lung surfactant function. Lung surfactant is a mixture of lipids and proteins that lines the alveolar region. The surfactant layer reduces the surface tension of the alveolar fluid, thereby playing a crucial role in lung stability. Due to their small size, particulates in e-cigarette vapor can penetrate the deep lungs and come into contact with the lung surfactant. The current study sought to examine the potential adverse effects of e-cigarette vapor and conventional cigarette smoke on lung surfactant interfacial properties.

**Methods:**

Infasurf^®^, a clinically used and commercially available calf lung surfactant extract, was used as lung surfactant model. Infasurf^®^ films were spread on top of an aqueous subphase in a Langmuir trough with smoke particulates from conventional cigarettes or vapor from different flavors of e-cigarettes dispersed in the subphase. Surfactant interfacial properties were measured in real-time upon surface compression while surfactant lateral structure after exposure to smoke or vapor was examined using atomic force microscopy (AFM).

**Results:**

E-cigarette vapor regardless of the dose and flavoring of the e-liquid did not affect surfactant interfacial properties. In contrast, smoke from conventional cigarettes had a drastic, dose-dependent effect on Infasurf^®^ interfacial properties reducing the maximum surface pressure from 65.1 ± 0.2 mN/m to 46.1 ± 1.3 mN/m at the highest dose. Cigarette smoke and e-cigarette vapor both altered surfactant microstructure resulting in an increase in the area of lipid multilayers. Studies with individual smoke components revealed that tar was the smoke component most disruptive to surfactant function.

**Conclusions:**

While both e-cigarette vapor and conventional cigarette smoke affect surfactant lateral structure, only cigarette smoke disrupts surfactant interfacial properties. The surfactant inhibitory compound in conventional cigarettes is tar, which is a product of burning and is thus absent in e-cigarette vapor.

## Background

An e-cigarette is a battery-powered device that delivers nicotine by heating a solution, commonly called e-liquid. E-liquids generally contain nicotine, humectants such as propylene glycol and glycerine, and flavorings. While the components of e-cigarettes might differ based on the producer, e-cigarettes generally contain a cartridge, which holds the e-liquid, an atomizer that serves to vaporize the e-liquid, a battery, and an LED light that illuminates during inhalation. E-cigarettes are becoming increasingly popular in the United States, especially among the younger generation. As of 2014, more than 20% of adults between the ages of 18–24 had tried an e-cigarette at least once [1]. In addition, a survey in 2015 revealed that 16% of high school students have used an e-cigarette at least once in the past 30 days, an increase of more than 10 fold compared to 2011 [2].

The increasing popularity of e-cigarettes has raised concerns regarding their safety. Due to their small size, particles in e-cigarette vapor are capable of penetrating the alveolar region of the lungs [3, 4]. In their path to the deep lungs, vapor particulates come into contact, directly or indirectly, with a variety of cells. As such, a number of recent studies have focused on investigating the potential toxic effects of e-cigarette vapor to cells of the upper and lower airways. While in almost all cases the toxicity of e-cigarette vapor has been shown to be less than the smoke from conventional cigarettes, toxicity from e-cigarette vapor has been reported depending on the cell line, e-liquid flavoring, and dose. Studies on the effects of e-cigarette vapor on bronchial epithelial cells, have shown a range of effects from little to no toxicity [5, 6] to loss of cell viability and oxidative and xenobiotic stress [7–9]. Similarly, studies on cells of the alveolar region have reported cytotoxicity depending on the dose and flavoring of the e-cigarettes used [10]. While studies on e-cigarette effects on pulmonary cells are undoubtedly important in understanding the potential adverse health effects of e-cigarette vapor, another potential aspect of e-cigarette toxicity, its potential adverse effects on lung surfactant function, has received far less attention.

Pulmonary surfactant is a thin fluid layer, which covers the alveolar region of the lungs. This surfactant layer is produced and secreted by alveolar type II cells and serves to reduce the surface tension of the alveolar fluid, thereby reducing the energy required to inflate the lungs and preventing alveolar collapse [11]. Surfactant interfacial properties play an important role in lung stability. Pulmonary surfactant deficiency leads to increased surface tension in the alveolar region, resulting in decreased lung compliance, impaired gas exchange, and alveolar collapse [12]. Elevated surface tension values in the alveoli and/or changes in surfactant composition have been reported in a number of diseases such as asthma [13], pneumonia [14], chronic obstructive pulmonary disease (COPD) [15], and respiratory distress syndrome [12, 16]. Lining the deep lungs, lung surfactant comes into direct contact with all particles that reach the alveolar region. While a number of recent studies have shown that small nano and sub-micron particles inhibit surfactant function in vitro [17–20], currently there is no information on how e-cigarette vapor might alter surfactant interfacial properties. In fact, even the effect of smoke from conventional cigarettes on lung surfactant interfacial properties are not well understood. It has been shown that cigarette smoke can exert deleterious effects on the function of surfactant models and extracts [21–24]; however, a comprehensive understanding of the components of cigarette smoke that might inhibit surfactant function is still lacking.

In the current study, the effects of e-cigarette vapor, generated from various flavors of e-liquids, as well as smoke from conventional cigarettes, on the interfacial properties and lateral structure of calf lung surfactant was investigated. It was found that while e-cigarette vapor and cigarette smoke both alter surfactant structure, only cigarette smoke disrupts surfactant interfacial properties. E-cigarette vapor, regardless of the flavoring, did not inhibit surfactant function. Studies with individual cigarette smoke components revealed that the insoluble particles in smoke (i.e. tar) are most disruptive to surfactant function. These results suggest that e-cigarettes, unlike conventional cigarettes, do not induce deleterious effects on lung surfactant interfacial properties.

## Methods

### Commercial reagents

Infasurf^®^ (lot: 112,809,225) was a generous gift from ONY Inc. (Amherst, NY). All organic solvents used in these studies were purchased from Fisher Scientific (Hampton, NH). Purified water used for all the experiments was obtained from an ELGA PURELAB Classic water purifier (High Wycombe, UK) and was used with a resistivity of 18.2 MΩ·cm. Unflavored, as well as berry- and mint-flavored e-cigarettes (all at 2.4% nicotine content) were purchased from Blu (Charlotte, NC). Conventional research cigarettes (1R6F) were purchased from the University of Kentucky Center for Tobacco Products (Lexington, KY). Glass fiber filters used to capture tar were purchased from EMD Millipore (Billerica, MA). Nicotine, isoprene, and acetaldehyde were all purchased from Fisher Scientific.

### Tensiometric studies

Interfacial experiments were carried out using a KSV NIMA Langmuir-Blodgett (LB) trough (Biolin Scientific, Finland). This apparatus was equipped with a Langmuir trough (364 mm × 75 mm × 4 mm, effective surface area = 243 cm^2^, subphase volume = 180 mL) made of hydrophobic polytetrafluoroethylene (PTFE), with a 20 mm × 56 mm × 60 mm dipping well. The trough was also equipped with two Delrin barriers, which enabled symmetric compression and expansion of the surface. Purified water was used as subphase for all studies. Our observations, as well as those reported by others [17], indicate that using water instead of buffers does not cause detectable changes in the surface pressure isotherm of Infasurf®.

For surface pressure isotherm measurements, purified water was poured in the trough, and was given 10 min to equilibrate at room temperature (24 ± 1 °C). Surface tension was measured in real-time using a platinum Wilhelmy plate (width: 19.62 mm, height: 38 mm, thickness: 0.10 mm), which yielded a value of 72.8 ± 0.3 mN/m as the surface tension of pure water. Since Infasurf^®^ samples were provided as surfactant suspensions in saline, samples needed to be extracted for tensiometric experiments. This was accomplished using the methods of Zhang and colleagues [25]. In brief, Infasurf^®^ lipids were extracted following the methods of Bligh and Dyer [26], the lipids were then dried under nitrogen gas and dissolved in chloroform at a concentration of 1 g/L. Approximately 30 μL of the resulting solution was spread at the air-water interface using a Hamilton micro-syringe (Hamilton company, Reno, NV) until an initial surface pressure of 20 mN/m was reached. Twenty minutes were given for the organic solvent to evaporate. The surface pressure of surfactant films was then recorded in real-time while the surface was compressed using the Delrin barriers. A barrier speed of 270 mm/min, which was the highest barrier speed available on the instrument, was used to mimic the fast compression in the lungs during exhalation. Surface pressure was calculated by subtracting the surface tension of pure water from the surface tension measured by the device after the lipids were added. Surface pressure was recorded upon surface compression and was plotted as a function of surface area to generate surface pressure isotherms.

For experiments with electronic or conventional cigarettes, different volumes of cigarette smoke or e-cigarette vapor were bubbled in the subphase prior to spreading the subphase in the trough. To this aim, the cigarettes were connected to a 150 mL syringe, the plunger of the syringe was then pulled back to create a vacuum and gather the smoke or the vapor, which was then bubbled in the subphase inside a covered beaker. Using this procedure, six drawings of the plunger (total of 900 mL) were required to completely “smoke” one conventional (1R6F) cigarette. This volume was used as the highest dose for both conventional cigarettes and e-cigarettes. Two lower smoke or vapor volumes of 90 mL and 9 mL were also used to study dose effects. For control experiments, 900 mL of clean air was used instead of smoke. Experiments with nicotine, acetaldehyde, and isoprene were performed similarly, except that each chemical was injected in the subphase prior to adding the surfactant. The amount of chemicals injected were equivalent to the amount reported in the smoke of one 1R6F cigarette as indicated in the certificate of analysis provided by the University of Kentucky Center for Tobacco Products: 721 ± 107 μg for nicotine, 522 ± 69 μg for acetaldehyde, and 320 ± 101 μg for isoprene.

**Fig. 1:**
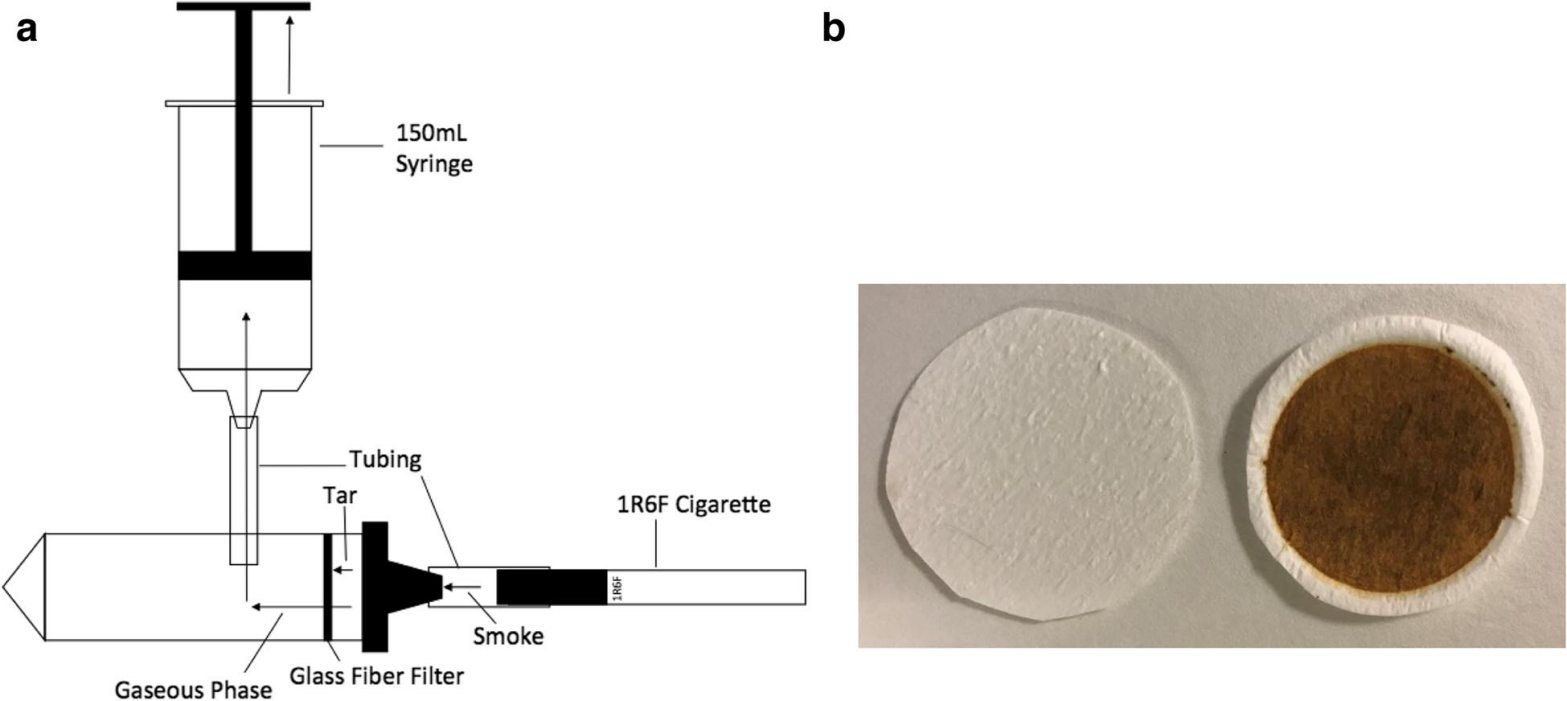
A custom-made setup was used for the entrapment of smoke particulates (tar) on filters. **a** Schematic of the setup and **b** Comparison between the glass fiber filters before (left) and after the entrapment of smoke particulates (right)

For experiments with tar, the particulate matter from burning research cigarettes was gathered on a filter pad. To this aim, a simple, custom-made experimental setup was built (Fig. 1a). In brief, a glass fiber filter (EMD Millipore, Model: AP1504700, pore size = 1 μm, particle retention = 0.2–0.6 μm) was cut to size and placed into the lid of a 50 mL conical tube. Then, a cigarette was lit and connected to the lid via tubing. A syringe was connected to the side of the tube and the plunger of the syringe was pulled to mimic smoking. The smoke was passed through the filter resulting in the entrapment of solid particles on the filter. The presence of tar on the filter was visible after the procedure (Fig. 1b). When the entire cigarette was “smoked” down to the filter, the glass fiber filter pad was removed with tweezers. The filter pad was then placed in a 50 mL beaker and 1 mL of acetone was added to the filter to extract the tar. The filter was then removed and the tar/acetone mixture was added to a 200 mL glass bottle, filled with 180 mL of deionized water and was shaken to evenly disperse the tar into the subphase. This subphase was then poured into the Langmuir trough and the isotherms were recorded as explained above. All tensiometric experiments with e-cigarette vapor, cigarette smoke, smoke components (including tar), and controls were performed in triplicates.

### Surfactant deposition on solid substrate for atomic force microscopy (AFM)

Langmuir-Blodgett deposition was used to deposit lipid monolayers on solid substrates for AFM imaging. For these experiments, a hydrophilic cover glass (VMR^®^, Randor, PA) made of borosilicate with the thickness of 0.13 mm and diameter of 32 mm was used as the substrate. The cover glass was washed using acetone, ethanol, and purified water and dried carefully prior to each experiment. After washing and drying, the cover glass was put in the dipper sample holder, and the dipper position was zeroed when the cover glass was barely touching the subphase. The dipper was then lowered until it was fully immersed in the subphase. The Infasurf^®^ solution was then spread on the subphase. At the desired surface pressure values (20 mN/m, 30 mN/m, 40 mN/m, and 50 mN/m), the cover glass was pulled up with the speed of 1 mm/min allowing for the deposition of the surfactant film while the barriers kept the surface pressure constant. The same procedure was performed for experiments with e-cigarette vapor and cigarette smoke, except that vapor or smoke were bubbled in the subphase prior to the addition of the surfactant. For experiments with cigarette smoke, deposition was performed at 20 mN/m, 30 mN/m, and 40 mN/m as these samples were unable to reach a surface pressure of 50 mN/m. For each surface pressure, 2 to 3 samples were examined for control, e-cigarette vapor, and cigarette smoke. On each sample, the surface area was monitored with large scans on typically 4 to 5 locations. If all locations showed similar surface features, an additional smaller high resolution scan was then taken. Analysis was done on the small high resolution scans and double-checked by running two of the larger scans.

### AFM procedure and image analysis

Surfactant lateral structure before and after exposure to cigarette smoke and e-cigarette vapor was examined by AFM Agilent 5500LS isolated from environment via passive and active isolation stages in an acoustic chamber. The AFM scans were performed using AC or tapping mode, which minimizes negative impacts of tip to surface collisions. Extended tip to surface contact have been shown to cause lipids to adhere to the tip, which in turn will bind and drag other lipids on the surface around, skewing the scan data. A 75 kHz Silicon tip (k = 2.7 N/m) was used for all imaging. Multiple preliminary AFM scans were taken in 10 μm^2^ scan areas on each sample to ensure sample uniformity and quality. These images are not reported because scans were taken at lower resolution. Once the sample uniformity and quality was confirmed, high resolution scans of 2 μm^2^ scan area were taken and recorded for each sample. All images were post-processed using Gwyddion^®^, which is an open-source analysis software.

Post processing of raw AFM data included two sets of processes, which were conducted precisely in the same manner for each sample to eliminate any systematic errors. The first processing was to translate the raw data into high quality topological images, a common practice in AFM analysis. The second was to further process the data for statistical analysis of the surface lipid formations. Topological AFM images were produced by the following process. First, the raw data was leveled by means of linear plane subtraction, which removes sample tilt. This was followed by a 4th order polynomial background fit and subtraction, which removes non-linearity cause by the movement of the scan head. Next, a 4th order polynomial row alignment was done to account for any drift while scanning. Finally, horizontal scars/strokes were marked using width, length and root mean square threshold values. Once these scars were marked, a Laplace transform was applied to correct the data. This last step accounts for any random noise or vibration that was coupled into the system during scans. These post-processing steps result in eliminating all artificial and systematic errors from the raw data. The corrected data was then saved as image files.

### Statistical analysis

Comparison of maximum surface pressure values and differences between the height and surface area coverage of AFM microstructures was performed using unpaired t-test using the GraphPad Prism software package (La Jolla, CA, USA). Statistical analysis on AFM raw data was performed using the Gwyddion^®^ software package. These analyses were performed to compare the height of the grains caused by ordered domains or multilayers and the surface area covered by these structures. First, grains were marked with a combination of segmentation, threshold, and watershed techniques. The intersection of the results from these three techniques defined the final grain, while all other data that were not marked by grain detection were removed. Then, the Gwyddion^®^ software was used to find the height and surface area coverage of the grains. For all experiments, data were reported as mean ± standard deviation and results were considered significant at *P* ≤ 0.05.

## Results

### Effects of e-cigarette vapor and cigarette smoke on surfactant interfacial properties

**Fig. 2:**
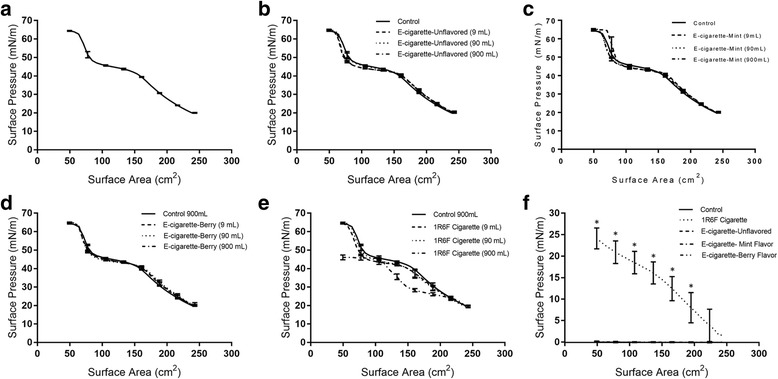
Surface pressure vs. surface area isotherms of Infasurf^®^ before and after exposure to various amounts of e-cigarette vapor and cigarette smoke. **a** Control (exposed to clean air), (**b**) exposed to unflavored e-cigarettes, (**c**) exposed to e-cigarettes with mint flavoring, (**d**) exposed to e-cigarettes with berry flavoring, (**e**) exposed to smoke from 1R6F conventional cigarettes, and (**f**) surface pressure vs. surface area isotherms of 900 mL of vapor or smoke bubbled in the subphase with no surfactant present (note that except for 1R6F cigarette all lines are close to zero), significant increase in surface pressure compared to control (using a *p*-value of 0.05) is shown by *

Surfactant interfacial studies were performed using Infasurf^®^ as the lung surfactant model. Infasurf^®^ is a calf lung surfactant extract with well-characterized interfacial properties, which is used in surfactant replacement therapy [16, 27]. The interfacial properties of Infasurf^®^ before and after exposure to e-cigarette vapor and cigarette smoke were characterized using a Langmuir-Blodgett trough equipped with a Wilhelmy plate. Infasurf^®^ films were spread on top of an aqueous subphase and were then symmetrically compressed to mimic the compression of the alveoli. Surface tension was measured in real-time allowing for the development of surface tension vs. surface area plots, which are generally reported as surface pressure (surface pressure = surface tension of pure subphase – surface tension of the subphase in the presence of surfactant). The surface pressure isotherm of Infasurf^®^, in the absence of smoke or vapor (control), is shown in Fig. 2a. In the surface pressure range of 20 mN/m to 40 mN/m, compression of Infasurf^®^ films results in an almost linear increase in surface pressure. In this surface pressure range, Infasurf^®^ films demonstrate distinct liquid-expanded (LE) and liquid ordered (LO) phases. Unsaturated lipids primarily localize in the LE phase, while the majority of saturated lipids are localized in LO phases [25, 28, 29]. In the range of 40 mN/m to 50 mN/m, the rate of increase in surface pressure is significantly reduced, in this range the unsaturated lipids collapse, leaving the surface highly enriched in saturated lipids and changing the structure of the film from a monolayer to a multilayer [25, 28, 29]. Compression beyond 50 mN/m resulted in a rapid and exponential increase in surface pressure until surfactant collapse was reached at a surface pressure of 65.2 ± 0.4 mN/m as evidenced by a plateau in surface pressure. The surface pressure isotherm of Infasurf^®^ reported in the current study is in good agreement with previously published surface pressure isotherms for this surfactant [17, 18, 25, 28–30].

Studies on the effects of e-cigarette vapor on Infasurf^®^ interfacial properties was performed using unflavored, as well as berry- and mint-flavored e-cigarettes. The berry and mint flavors were chosen due to their reported popularity among e-cigarette users [31]. The effects of e-cigarette vapor were compared with the effects of smoke from a cigarette (research cigarette 1R6F, University of Kentucky Center for Tobacco Research Products). The effect of e-cigarette vapor on Infasurf^®^ interfacial properties was examined by bubbling various amounts of vapor (9 mL, 90 mL, and 900 mL) in the subphase. This was accomplished by connecting the e-cigarettes to a syringe, which was used to create a vacuum to gather the vapor and bubble it in the subphase. The highest vapor volume of 900 mL was the amount of smoke generated by “smoking” one complete 1R6F cigarette using this setup. Exposure to e-cigarette vapor did not significantly affect the surface pressure isotherm of Infasurf^®^, regardless of the flavor of the e-liquid (Fig. 2b-d). In contrast, a significant surfactant inhibition at the highest cigarette smoke volume was observed (46.1 ± 1.3 mN/m compared to 65.1 ± 0.2 mN/m for control). This effect was dose-dependent and only occurred at the highest smoke volume of 900 mL (Fig. 2e).

Surface active components are known to be disruptive to lung surfactant function because of their ability to compete with surfactant for space at the air-water interface [32–34]. Thus, to understand whether the effects of cigarette smoke on surfactant function is due to its surface activity, e-cigarette vapor from all e-liquid flavors and smoke from 1R6F cigarettes were bubbled in the subphase at the highest volume (900 mL), the subphase was then compressed in the absence of surfactant to measure the surface activity of the soluble components of vapor and smoke. E-cigarette vapor, regardless of the e-liquid flavor, did not significantly affect the surface pressure of water, leading to near zero surface pressure values upon compression. However, cigarette smoke was highly surface active, resulting in a surface pressure of 25.1 ± 3.5 mN/m at the end of compression (Fig. 2f), which suggests a correlation between the surface activity of smoke and its deleterious effects on surfactant function. Taken together, surfactant interfacial studies demonstrate that at similar doses cigarette smoke, unlike e-cigarette vapor, is detrimental to Infasurf^®^ surface activity and that the disruptive effects of cigarette smoke on Infasurf^®^ are likely due to the presence of surface active components that interfere with surfactant function.

### Effects of e-cigarette vapor and cigarette smoke on surfactant lateral structure

The lateral organization of surfactant molecules is crucial in their ability to reach low surface tension (i.e. high surface pressure) values [25]. As mentioned above, compression of Infasurf^®^ results in phase separations as well as the collapse of unsaturated lipids into multilayers. The height difference between the ordered and disordered phases, as well as multilayers and the monolayer, provides a unique lateral organization to the surfactant [17, 25, 29]. AFM imaging was used to study the effects of e-cigarette vapor and cigarette smoke on surfactant lateral organization. Since none of the e-cigarettes significantly altered Infasurf^®^ interfacial properties, these studies were performed with unflavored e-cigarettes. For these studies, surfactant films were deposited on solid substrates at surface pressure values of 20, 30, 40, and 50 mN/m, using Langmuir-Blodgett deposition, and were then imaged using AFM.

**Fig. 3:**
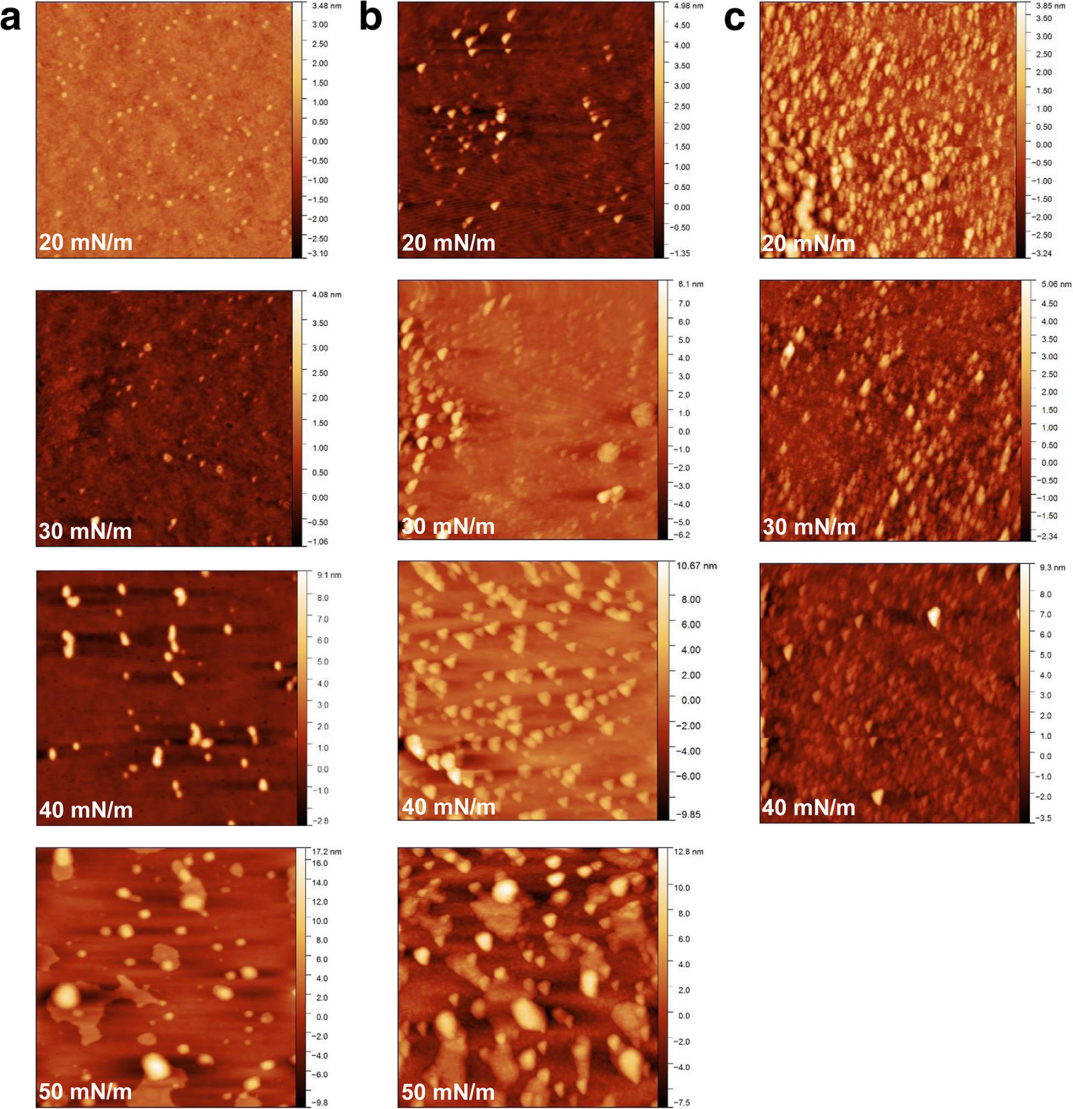
Infasurf^®^ surface topology at various surface pressure values (20, 30 mN/m, 40 mN/m. and 50 mN/m) as examined by atomic force microscopy after exposure to 900 mL of (**a**) clean air, (**b**) e-cigarette vapor, and (**c**) conventional cigarette smoke. Note that Infasurf^®^ collapsed at 46.1 ± 1.3 mN/m after exposure to cigarette smoke; therefore surface topography could not be examined at 50 mN/m for this sample

**Fig. 4:**
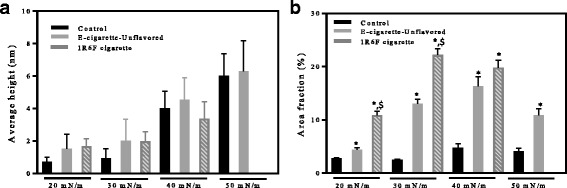
Features of the Infasurf^®^ surface structures at various surface pressure values before and after exposure to e-cigarette vapor (unflavored) and cigarette smoke as determined by atomic force microscopy: (**a**) average height and (**b**) average surface area covered by the structures. Cigarette smoke resulted in an increase in area fraction, which was significantly increased compared to control (shown by *), and in some cases to surfactant exposed to e-cigarettes vapor (shown by $). A *p*-value of 0.05 was used for all analysis

AFM studies of pure Infasurf^®^ films showed surfactant phase separation, resulting in a hill and valley structure, as previously reported in the literature (Fig. 3a). In these images, brightness corresponds to the height of the structures. At surface pressure values of less than 40 mN/m, Infasurf^®^ films showed distinct LE and LO phases. The difference in the order and the height of saturated lipids in the LO phase compared to unsaturated lipids in the LE phase, results in LO phase structures being taller compared to the LE phases as observed in Fig. 3a at 20 mN/m and 30 mN/m and previously reported for other surfactants [35–37]. At a surface pressure of approximately 40 mN/m, the surfactant undergoes an enrichment phase in which the disordered phases start to collapse into multilayers leaving a monolayer enriched in saturated lipids. Thus, at 40 mN/m and 50 mN/m the hills correspond to multilayers while the valleys correspond to the ordered phases. The collapse of the monolayers into multilayers is best evidenced by the increase in the height of the surface structures. Before the formation of multilayers the average height of the surface structures was 0.713 ± 0.003 nm (at 20 mN/m) and 0.947 ± 0.006 nm (at 30 mN/m). However, the average heights increased to 4.019 ± 1.032 nm at 40 mN/m and 6.015 ± 1.357 nm at 50 mN/m marking the presence of multilayers that increase in height as the surface is further compressed (see Fig. 4 for detailed quantification). It should be noted that the presence of a cholesterol-rich, tilted-condensed (TC) phase inside the LO phases has also been reported [25, 29]. This phase is not readily apparent in the AFM images in Fig. 3. This is likely due to the fact that the AFM scans in the current study are “zoomed-in” 2 × 2 μm scans, while the majority of literature reports on Infasurf^®^ topology are 20 × 20 μm scans. However, the trends regarding the increase in domain heights and the height values are in close agreement with the literature [25]. In summary, AFM studies reveal a hill and valley lateral structure caused by lipid phase separation and the formation of multilayers as the surfactant is compressed.

Exposure to e-cigarette vapor or cigarette smoke did not significantly change the height of the LO phases or the multilayers, but significantly increased their surface area compared to control (Fig. 3, middle and right columns). In each case, surfactant topology followed the same trend as pure Infasurf^®^ films with the size and height of LO phases increasing upon compression and multilayers forming at high surface pressure values. In the case of cigarette smoke, the size and height of the structures could not be investigated at 50 mN/m; this is because the surfactant collapsed at 46.1 ± 1.3 mN/m. The height and surface area fraction of Infasurf^®^ structures before and after exposure to e-cigarette vapor and cigarette smoke is presented in Fig. 4. While exposure did not significantly alter the height of the structures (Fig. 4a), a significant increase in the area covered by the domains could be observed particularly after exposure to cigarette smoke. For example, at the surface pressure of 20 mN/m, LO domains covered 10.9 ± 0.7% of the surface, this value was significantly larger than the area covered by the LO phases in case of both e-cigarettes (4.4 ± 0.4%) and control (2.8 ± 0.1%) at the same surface pressure. A similar trend continued through the compression at all surface pressure values (Fig. 4b). These observations demonstrate that while the hill and valley lateral structure of Infasurf^®^ was not abrogated by exposure to e-cigarette vapor or cigarette smoke, exposure resulted in a notable increase in the surface area of the hills, indicating a significant change in surfactant lateral organization.

### Effects of cigarette smoke components on surfactant interfacial properties

**Fig. 5:**
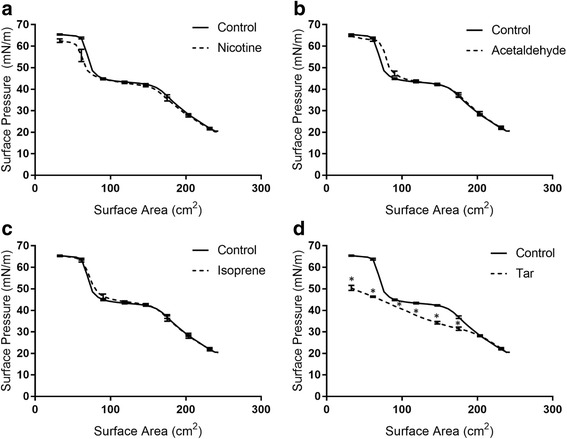
Surface pressure vs. surface area isotherms of Infasurf^®^ before and after exposure to the most abundant components in smoke from 1R6F cigarettes, (**a**) nicotine, (**b**) acetaldehyde, (**c**) isoprene, and (**d**) tar. The amount of each component is equal to the amount in one complete cigarette according to the certificate of analysis of 1R6F cigarettes: 721 μg for nicotine, 522 μg for acetaldehyde, and 320 μg for isoprene, while the experiment with tar was performed with the particulate matter gathered on glass fiber filters from one complete cigarette. Significant increase in surface pressure compared to control (using a *p*-value of 0.05) is shown by *

Given the drastic effects of smoke from conventional cigarettes on Infasurf^®^ interfacial properties, further experiments were performed to identify the smoke component that is most damaging to surfactant interfacial properties. The smoke components of 1R6F cigarettes are provided in the certificate of analysis by the manufacturer (University of Kentucky, Center for Tobacco Research Products). The three components with the highest concentration are nicotine (721 ± 107 μg/cigarette), acetaldehyde (522 ± 69 μg/cigarette), and isoprene (320 ± 101 μg/cigarette). Each one of these components was added to the subphase, in the amount corresponding to one full cigarette, and Infasurf^®^ surface pressure vs. surface area isotherm was obtained in their presence to identify the components in cigarette smoke that causes surfactant inhibition. It should be noted that cigarette smoke contains many different chemicals and it is not practical to add each component to the subphase; however, the three components mentioned above are the ones with the highest concentration. All other non-gaseous components of cigarette smoke are at least one degree of magnitude lower in concentration, with the next highest being acrolein (43 ± 14 μg/cigarette). Interestingly, none of nicotine, acetaldehyde, and isoprene significantly altered Infasurf^®^ interfacial properties (Fig. 5a-c). Nicotine caused a very minor reduction in the maximum surface pressure (62.6 ± 0.8 mN/m compared to 65.1 ± 0.2 mN/m for control), which was quite different compared to the drastic reduction in surface pressure observed in the presence of cigarette smoke. The surface pressure isotherm in the presence of acetaldehyde and isoprene were also very similar to pure Infasurf^®^. In an effort to identify the component that causes surfactant inhibition, the particulate matter caused by burning cigarettes (tar) was gathered on a filter and added to the subphase in a separate experiment. Interestingly, the addition of tar, in the amount corresponding to one cigarette, caused significant surfactant inhibition (Fig. 5d). The maximum surface pressure in the presence of tar was 50.4 ± 1.2 mN/m; this value was comparable to the maximum surface pressure observed for Infasurf^®^ after exposure to cigarette smoke (46.1 ± 1.3 mN/m) and very different from pure Infasurf^®^ (65.1 ± 0.2 mN/m). Taken together, these experiments demonstrate that while nicotine, acetaldehyde, and isoprene, all of which are major components of smoke, do not deteriorate the surface activity of Infasurf^®^, tar is highly disruptive to surfactant interfacial properties and is likely the most disruptive component to surfactant in cigarette smoke.

## Discussion

With the increasing use of e-cigarettes, research into their potential pulmonary toxicity has intensified. While an increasing number of studies have focused on the potential cytotoxicity of e-cigarettes, the interactions of e-cigarette vapor with the pulmonary surfactant have received less attention and remain largely unknown. The current study aimed to fill this gap of knowledge and provide an understanding and comparison of the potential disruptive effects of e-cigarette vapor and cigarette smoke on lateral structure and interfacial properties of the pulmonary surfactant.

Studies using calf lung surfactant revealed that e-cigarette vapor does not affect surfactant interfacial properties regardless of the e-liquid flavoring (Fig. 2). The lack of effects from e-cigarette vapor on Infasurf^®^ can be explained by considering the components in e-cigarette vapor. The primary components of e-cigarette vapor are propylene glycol, glycerol, and nicotine [38, 39]. Propylene glycol and glycerol are both hydrophilic and are thus likely to remain in the aqueous subphase and not disturb the lipid film at the air-water interface. On the other hand, nicotine has a very minor effect on surfactant properties as shown in Fig. 5a. Thus, none of the components of e-cigarette smoke are expected to cause significant disruptions to surfactant films. To the best of our knowledge, there has only been one previous study on the interactions of e-cigarette vapor with lung surfactant [38]. In this study, Davies and colleagues used a mixture of dipalmitoyl phosphatidylcholine, phosphatidyl glycerol, and phosphatidic acid (DPPC/POPG/PA, 69/20/11, *w*/w/w) to mimic lung surfactant, and reported a slight reduction (~10 mN/m) in the surface pressure of this model after exposure to e-cigarette vapor [38]. The potential mechanisms of this detrimental effect were proposed to be nicotine penetration in the surfactant monolayer, lipid peroxidation by free radicals in the vapor, and/or hydrolysis of surfactant phospholipids by nitrosamines in smoke [38]. In the current study, pure nicotine showed a very minor effect on the surface pressure isotherm of Infasurf^®^. While free radicals [40] and nitrosamines [39] have both been reported in e-cigarette vapor, any potential effects from these components on Infasurf^®^ in the current study was minor. It should be noted, however, that the effects of these chemicals on a complex surfactant such as Infasurf^®^ might be very different compared to the simpler model used by Davies and colleagues [38].

In contrast to e-cigarette vapor, cigarette smoke significantly disrupted surfactant interfacial properties. The disruptive effects of e-cigarette vapor and cigarette smoke correlated with their surface activity. E-cigarette vapor, which did not affect the surface pressure, was not surface active, while cigarette smoke was highly surface-active (Fig. 2e). This correlation between surface activity and surfactant disruption suggests that surfactant inhibition by smoke occurred through the competitive adsorption mechanism [33]. Based on this mechanism, surface active components, in this case from cigarette smoke, are capable of adsorption to the air-water interface and compete with surfactant molecules for space. Adsorption of non-surfactant molecules hinders surfactant adsorption at the air-water interface and interferes with the enrichment of the surface with highly saturated lipids, thereby inhibiting the ability of the surfactant to reduce the surface tension (i.e. increase the surface pressure). A similar mechanism has been shown to be the underlying principle for surfactant inhibition by albumin [34, 41, 42]. Albumin is a surface-active protein and can reach surface pressure values higher than 30 mN/m upon compression [34, 41]. Due to its surface-activity, albumin adsorbs to the air-water interface, interfering with surface adsorption of surfactant molecules and eventually leading to a reduction in the maximum surface pressure achievable by surfactant [34, 41, 42].

This competitive adsorption mechanism is further supported by AFM images. The plateau in Infasurf^®^ surface pressure at ~40 mN/m is the start of the process where unsaturated lipids collapse into multilayers, leaving a surface enriched in highly saturated lipids [25, 29]. This process was observed in the current study where the height of the surfactant structures changed from <1 nm at surface pressure values of 20 and 30 mN/m to 4 and 6 nm at surface pressure values of 40 and 50 mN/m, respectively, due to the presence of the multilayers. Addition of e-cigarette vapor or cigarette smoke did not significantly affect the height of these structures, suggesting that the multilayer formation process was not affected. However, the surface area covered by unsaturated multilayers highly increased as a result of exposure to vapor and smoke. This increase in surface area was quite drastic in the case of cigarette smoke (Fig. 4b). The significant increase in the area of unsaturated lipids consequently hinders the enrichment of the surface by saturated lipids, leaving a surface with a high level of unsaturated lipids that cannot reach high surface pressure values. Particles in cigarette smoke have been reported to have a mass median diameter of 380 nm [43]. Thus, given the average height of the AFM structures, it is unlikely that a large portion of smoke particles have directly penetrated the air-water interface. It appears more likely that water-soluble components from the smoke have adsorbed to the air-water interface, likely partitioning with unsaturated lipids, resulting in an increase in the surface area of unsaturated multilayer phase. A similar phenomenon has been reported for albumin molecules that partition into disordered lipid phases in bovine lipid extract surfactant [34].

While exposure to cigarette smoke is known to alter the level of surfactant lipids and proteins in animals [22, 44] and in humans [45], the effects of cigarette smoke on surfactant interfacial properties remain understudied. Early studies with bronchoalveolar lavage (BAL) have shown that cigarette smoke can affect surfactant function and respreadability [21, 22]. However, as noted by Bringezu and colleagues [23], interfacial studies with BAL are difficult due to the large variability associated with the extraction of BAL and isolation of surfactant and there is a need for more mechanistic studies. To the best of our knowledge, only two detailed mechanistic studies exist on the surfactant inhibitory effects of cigarette smoke [23, 24], both using environmental tobacco smoke (ETS), a combination of smoke from the smoldering cigarette and the smoke inhaled by the smoker (note that only the latter is being examined in the current study) [46]. In one study, ETS was mixed with a (DPPC/POPG/PA, 69/20/11, *w*/w/w) surfactant model, resulting in slight changes in surfactant respreading and maximum surface pressure values [23]. These effects were attributed to smoke particulates removing the unsaturated POPG to the subphase resulting in a highly saturated surfactant which cannot efficiently respread after compression [23]. While this mechanism seems quite plausible for the DPPC/POPG/PA model, it is less likely to be significant for Infasurf^®^, as Infasurf^®^ only has 5% POPG [27] compared to 20% in the model of Bringezu and colleagues [23]. In addition, since saturated lipids are the driving force for reaching high surface pressure values, removal of unsaturated lipids should result in only minor effects in the ability of the surfactant to reach high surface pressure values; however, the surfactant inhibition caused by cigarette smoke in the current study is quite drastic. A second study on ETS effects on more complex, natural surfactants proposed a slightly different mechanism [24]. In this case, ETS exposure was shown to alter the lateral distribution of the porcine derived surfactant, Curosurf^®^, reducing the size of the ordered lipid domains and resulting in a surface that was enriched in unsaturated lipids and less effective in increasing the surface pressure [24]. The latter mechanism better aligns with the findings of the current study where cigarette smoke particles have resulted in an increase in the surface area of unsaturated multilayers.

Our tensiometric studies with the most abundant components in cigarette smoke clearly suggest that tar (i.e. the product of burning) is the main disruptive agent to surfactant interfacial properties (Fig. 5). These studies were performed with the components of highest concentration in cigarette smoke, based on the certificate of analysis of 1R6F cigarettes [47]. The smoke composition of 1R6F cigarettes generated by the University of Kentucky Center for Tobacco Reference Products closely mimics the smoke composition of other research cigarettes produced and analyzed by the same source and by others [48–50]. While we cannot rule out the presence of other surfactant inhibitory compounds in cigarette smoke, all other chemicals in cigarette smoke were at least one order of magnitude lower in concentration compared to those tested. The presence of a high amount of tar (as evidenced by Fig. 1b), suggests that perhaps modifications to the cigarette filters might be able to reduce some of these inhibitory compounds. These findings also explain why e-cigarette vapor was not detrimental to surfactant: e-cigarette is a result of e-liquid vaporization, but not burning. Little to no tar is expected from vaporization, which explains the lack of disruptive effects.

It should be noted that while the present study suggests that e-cigarette vapor does not directly affect the interfacial properties of lung surfactant, both e-cigarette vapor and cigarette smoke could impact lung surfactant function via indirect mechanisms. Such mechanisms could include protein/lipid oxidation and alterations in the expression or release of key surfactant components. E-cigarette vapor contains reactive free radicals [40] and reactive oxygen species [51, 52]. In addition, exposure to e-cigarette vapor has been shown to increase the expressions of genes involved in oxidative stress pathways of human bronchial epithelial cells [7]. While the downstream effects of such events on surfactant production and secretion are not yet known, it is quite plausible that exposure to reactive and oxidative species in e-cigarette vapor and increased oxidative stress might lead to oxidation of surfactant lipids and proteins and/or affect surfactant production or secretion. On the other hand, cigarette smoke has been shown to cause oxidative injury in type II alveolar cells [53, 54] and reduce the production and alter the secretion of surfactant phophospholipids by these cells [22, 55]. Thus, cigarette smoke is likely to inhibit surfactant function through both direct and indirect mechanisms while e-cigarette vapor might be capable of indirect surfactant disruption.

It is important to note some of the limitations of the current study and put the results in greater context. Here, exposure to smoke and vapor particulates was performed by bubbling the smoke and vapor in the subphase. This method has been previously used to study smoke cytotoxicity [55, 56] and was employed due to challenges in reproducible aerosol generation and quantification of the deposited particles, some of which have been addressed elsewhere [18, 57]. However, surfactant exposure to aerosols is a more physiologically-relevant exposure method and needs to be considered for future studies. Another limitation of the current study is that experiments were performed at room temperature; this is due to the fact that increased temperature reduces the size of surfactant domains, making them difficult to discern particularly at low surface pressure values. It should also be noted that the results presented in this study only focus on one aspect of potential e-cigarette toxicity. Thus, lack of surfactant disruption by e-cigarette vapor, does not suggest that e-cigarettes are safe. Increasing reports are emerging on the cytotoxicity, xenotoxicity, and inflammatory effects of e-cigarettes, which will help evaluate whether e-cigarette use will lead to other potential health effects. In addition, our study was focused on one brand and a limited number of e-liquid flavors and potential toxicity by other e-cigarettes cannot be ruled out. On the other hand, our results with conventional cigarettes further emphasize the association between cigarette use and respiratory toxicity. Changes in surfactant interfacial properties are associated with a number of respiratory diseases and can result in increased work of breathing and impaired gas exchange. While the effects of conventional cigarette smoke on surfactant production have been studied in the past [22, 44, 45], smoke effects on surfactant function and interfacial properties have received less attention and the results from this study help elucidate the disruptive effects of cigarette smoke on lung surfactant function and identify the component that is most harmful to surfactant interfacial properties.

## Conclusion

In summary, this study demonstrates that e-cigarette vapor does not inhibit the interfacial properties of calf lung surfactant despite causing minor changes in surfactant lateral structure. In contrast, cigarette smoke significantly inhibits surfactant interfacial properties. The disruptive effects of cigarette smoke are caused by tar, which is generated in the process of burning the tobacco, and are absent in e-cigarettes. These results are useful in evaluating the respiratory toxicity of both conventional and electronic cigarettes.
